# Muscles and the Media: A Natural Experiment Across Cultures in Men’s Body Image

**DOI:** 10.3389/fpsyg.2020.00495

**Published:** 2020-04-03

**Authors:** Tracey Thornborrow, Tochukwu Onwuegbusi, Sophie Mohamed, Lynda G. Boothroyd, Martin J. Tovée

**Affiliations:** ^1^School of Psychology, College of Social Science, University of Lincoln, Lincoln, United Kingdom; ^2^Department of Psychology, Durham University, Durham, United Kingdom; ^3^Department of Psychology, Northumbria University, Newcastle Upon Tyne, United Kingdom

**Keywords:** body image, muscularity, body ideals, cross-cultural, ethnicity

## Abstract

An increasing number of studies are evidencing relationships between the drive for muscularity and potentially harmful behavioral strategies, such as unhealthy dieting and steroid use amongst men in WEIRD (Western, Educated, Industrialized, Rich, Democratic) populations. As such Western appearance standards proliferate around the world via the media, men who live in other cultural contexts are also at risk of potentially negative effects from aspiring to the “muscular ideal.” However, few studies have explored these relationships in non-WEIRD populations. We investigated men’s body ideals and body image in two non-WEIRD, non-White populations, Uganda (Africa) and Nicaragua (Central America), and compared them with an ethnically diverse sample of men in the United Kingdom. We also examined whether socio-cultural factors including media and ethnicity, predicted the drive for muscularity and body change behaviors among our participants. Results showed that Ugandan men had the least desire for muscularity relative to men in the United Kingdom. Supporting the Tripartite model we found that media and peer influences significantly predicted the drive for muscularity, particularly among men from White British and Nicaraguan Miskitu ethnic groups. By contrast, Creole / Garifuna and Mestizo men from Nicaragua were more likely to want to increase muscularity relative to Black African men from Uganda. Overall, our findings support previous research in demonstrating that there are cultural differences in the kind of body men desire, and that men from WEIRD and non-WEIRD populations may experience similar pressures to aspire to and attain a muscular body type.

## Introduction

The often unrealistic appearance standards that proliferate Western media are believed to influence people’s body ideals. “Ideal” women are portrayed as “curvaceously thin,” with a low body weight but full breasts (Harrison, 2003), while “ideal” men have bodies with “worked-out” muscles and v-shaped torsos (Edwards et al., 2014). Both correlational and experimental studies have shown that even relatively short durations of exposure to the media’s body ideals can have a detrimental effect on women’s body image (Grabe et al., 2008). Less conclusive evidence suggests that media exposure is also associated with negative effects on men’s body image (Blond, 2008; Hausenblas et al., 2013) in similar but not identical ways (Barlett et al., 2008; Tamplin et al., 2018).

As the media become ever more pervasive, and Western appearance standards are broadcast to people around the world, the need for research that measures the media’s impact on body image and well-being beyond a White, Western demographic becomes more pressing. Studies show that even in non-WEIRD (that is Western, Educated, Industrialized, Rich, Democratic; see Henrich et al., 2010) populations with relatively recent exposure to Western media, television consumption is associated with slimmer female body ideals among both men and women (Jucker et al., 2017; Thornborrow et al., 2018; Boothroyd et al., 2019) and with disordered eating among women (Becker et al., 2002) and dieting behaviors (Boothroyd et al., 2016). While few studies have investigated media influence on men’s body image outside of a WEIRD demographic (Edwards et al., 2014), scant evidence suggests that men in non-WEIRD populations are also beginning to aspire to the muscular ideal body type (Holmqvist and Frisén, 2010) and experience body image concerns (Ricciardelli et al., 2007a; Xu et al., 2010).

Chronic media saturation of the physical and sociocultural environment means such idealized bodies proliferate people’s “visual diet” and become normalized (Boothroyd et al., 2012; Smirles and Lin, 2018). While not every individual who is exposed to media’s appearance ideals will experience negative effects, those who cognitively adopt these body ideals may be at greater risk than those individuals who do not (Nouri et al., 2011). This acceptance of, and aspiration toward, an external, socially constructed appearance standard is known as internalization (Thompson and Heinberg, 1999). While there is considerable evidence that among girls and women, internalization of media’s body ideals is associated with detrimental effects on body image and well-being (Grabe et al., 2008), there is less evidence of this relationship among men. Furthermore, the contributing factors, pathways and outcomes appear to be more varied, and are less well understood (Edwards et al., 2014).

As the societal body ideal for women is based on achieving and maintaining a low body weight, women’s body dissatisfaction is most often associated with a drive for a thinness (although not always, see Cramblitt and Pritchard, 2013). Conversely, the societal ideal body for men demands a developed musculature (particularly the upper body) and leanness to produce a “v” shaped torso. As a consequence men’s body concerns are predominantly associated with a drive for muscularity (Edwards et al., 2014), although a drive for thinness may be involved too (Pritchard and Cramblitt, 2014). Research evidence shows that men and adolescent boys are increasingly experiencing pressure to conform to the “muscular ideal” body type (Ricciardelli et al., 2007b; Waling, 2017). In the United States, 60% of adolescent boys reported engaging in muscle enhancing behaviors (Eisenberg et al., 2012). Furthermore, evidence suggests that in clinical diagnoses, the occurrence of extreme muscle enhancing behaviors are becoming as common among young men as severe weight control and purging behaviors are among young women (Murray et al., 2016; Karazsia et al., 2017; Lavender et al., 2017).

### The Drive for Muscularity

The drive for muscularity can be defined as the cognitive tension between an individual’s perception of their body as insufficiently muscular and the external, usually culturally-constructed muscular ideal that they believe they need to attain (Colman, 2015). A number of studies have identified associations of the drive for muscularity with actual media exposure, and with media internalization (Daniel and Bridges, 2010; Cramblitt and Pritchard, 2013; Pritchard and Cramblitt, 2014). However, although internalization in these studies significantly predicted men’s drive for muscularity, relationships with actual media exposure were weaker. This could be due to the way media exposure was measured or it could indicate that other sources of societal pressure also play a role in this relationship (Pritchard and Cramblitt, 2014). For example, men’s ideal body types may be more prevalent among peers or “specialist” groups such as athletes, than in mainstream media (Karazsia and Crowther, 2009).

While several studies have shown that the drive for muscularity in men is associated with body dissatisfaction and other negative psychological outcomes such as low self-esteem (Edwards et al., 2014; Girard et al., 2017), this is not always the case. For example, body builders or men who work out regularly may have a high drive for muscularity, but are also physically close to their ideal body and so may not experience body dissatisfaction (Tylka, 2011). Crucially however, even if body dissatisfaction is not present, there is strong evidence that a drive for muscularity is associated with potentially harmful body change behavioral strategies, including excessive weight training, steroid use, and unhealthy dieting behaviors (Edwards et al., 2014). This would suggest that a drive for muscularity is not qualitatively the same as the drive for thinness which is clearly associated with body dissatisfaction and disordered eating (McCreary and Sasse, 2000). Consistent with this hypothesis, men with body image disorders show a split between those who strive for thinness and those who seek to increase muscularity (McCabe and Ricciardelli, 2004; Adams et al., 2005; Barlett et al., 2008).

Most studies investigating drive for muscularity among men have sampled White men in Western populations (i.e., Australia, United States, and United Kingdom) (Ricciardelli et al., 2007b; Jung et al., 2010; Edwards et al., 2014). The limited number of studies which have sampled from outside this demographic have produced mixed findings. While Chinese males were less likely to value muscularity than United States men (Jung et al., 2010), another study found less differences between Asian American and European American men (Cheng et al., 2016). In the United Kingdom, Black and Asian men reported higher drive for muscularity than White men (Swami, 2016). Thus far, due to the low numbers of studies carried out, no consistent pattern relating to body image or drive for muscularity among non-White men in different cultural settings has been identified (Ricciardelli et al., 2007b).

### Sociocultural Influences on Men’s Body Image

Much of the research investigating sociocultural influences on body image has focused on media, likely because in Western contexts where most studies are carried out, mass media are the primary transmitters of societal appearance standards. However, studies in a number of countries suggest that other sociocultural influences, such as friends and family, may be more salient factors in shaping body ideals and influencing body image among adolescent boys and men (Karazsia and Crowther, 2009; Tylka, 2011; Stratton et al., 2015). For example, among Australian adolescent boys, comments from fathers were significantly associated with their exercise behaviors, and mothers were viewed as having a greater influence on the boys’ body image than other sociocultural factors (Ricciardelli et al., 2000). For Turkish adolescent boys, comments from male peers predicted appearance importance, and fathers’ attitudes influenced behaviors to attain a muscular ideal body (Dogan et al., 2018). Several other studies have found that boys were more likely to be influenced by appearance pressures from family, particularly in non-WEIRD populations (Mellor et al., 2008; McCabe et al., 2015).

The Tripartite Influence model (Thompson et al., 1999) is particularly useful for investigating predictors of body image related outcomes in non-WEIRD settings, where Western media (and thus media’s appearance ideals) may not be so pervasive, or where cultural values are traditionally transmitted predominantly via peers or family members. The model proposes that three main sociocultural influences, family, peers and the media, are largely responsible for conveying and maintaining appearance-related standards and messages. Such sociocultural messages can impact individuals’ body image and behaviors both directly, and indirectly through the processes of appearance comparison and internalization. The model has been used to predict body dissatisfaction among children (Rodgers et al., 2015), weight satisfaction and disordered eating among women (Markey, 2004), and body dissatisfaction and muscularity-enhancing behaviors among men (Karazsia and Crowther, 2009; Tylka, 2011; Stratton et al., 2015; Girard et al., 2017). However, there has been less use of a Tripartite model in non-WEIRD contexts, and research has predominantly focused on the female population.

In short, although increasing numbers of studies are evidencing relationships between the drive for muscularity and potentially harmful behavioral outcomes among men (Edwards et al., 2014), including some in non-White, non-WEIRD populations (Ricciardelli et al., 2007b), our understanding of the sociocultural influences, mechanisms and pathways involved is far from complete. As previously discussed, few studies have investigated men’s body image in non-WEIRD populations. Even fewer studies have explored ethnic differences in body image and body-related behaviors within such populations: There is often a tendency to culturally “umbrella” populations within single countries (Swami, 2016). Even within supposedly culturally homogenized Western nations, body ideals and the experience of body image may vary across ethnic groups (Yanover and Thompson, 2010; Cheng et al., 2016; Swami, 2016).

### The Present Study

The present study had two main aims: Firstly, we investigated men’s body ideals and body image in two non-WEIRD, non-White populations, Uganda (Africa) and Nicaragua (Central America), and compared them with an ethnically diverse sample of men in the United Kingdom. Secondly, we investigated whether media exposure and other sociocultural factors, including ethnicity, predicted the drive for muscularity and body change behaviors among our participants.

Here, we provide some demographic context for Uganda and Nicaragua, firstly to demonstrate that the populations from which we drew our samples are non-WEIRD, and secondly to avoid glossing over cultural differences that may not be expressed by using nationality as the cultural indicator (Holmqvist and Frisén, 2010).

Uganda is situated in East Africa and has a population of approximately 34.6 million people. Mean life expectancy is 63.7 years and 55% of the populations are under 18 years old (Ugandan Bureau of Statistics, 2014). Uganda is an ethnically diverse country, comprising of 56 distinct tribes. Approximately 17% of indigenous people belong to the leading ethnic group, the Buganda Kingdom. Although these ethnic groups speak different languages, English is the official national language of Uganda. In terms of education, in 2014 there was an 81% Net Enrolment Rate (NER) at primary school level, 32% enrolment at secondary level and 7% in higher or further education. The country is becoming more urbanized over time. This urbanisation is mirrored by an emerging middle-class population that is expanding at a faster rate than the total Ugandan population, and is developing a more “Westernized” lifestyle and outlook (Ayoki, 2012). Uganda’s Gini index is 39.5, 21.4% of the population are below the poverty line and gross national income per capita is approximately $2,400 (World Factbook, 2019). In Uganda’s capital Kampala, where we recruited participants for the current study, 68.1% of households own a television and 40.2% have access to the internet (Ugandan Bureau of Statistics, 2014), and it is likely that these statistics have since increased. Additionally, the majority of the Ugandan participants were recruited from a manufacturing company, where staff have access to the internet during work hours.

Nicaragua is situated in Central America and has an estimated population of just over 6 million. Over 48% of the population are under 25 years of age and life expectancy is 73.5 years (World Factbook, 2019). The population is ethnically diverse, although a vast majority identify as Mestizo (mixed Spanish European and indigenous background). Other ethnic groups include Creole and Garifuna, who identify as of predominantly Black Caribbean and Black African descent, and Miskitu, Ulwa and Sumu, who identify as indigenous people. Individuals are often of mixed heritage but will usually align themselves with one particular ethnic group. Although the official language is Spanish, on the Caribbean coast where the present research was carried out, many people speak English Creole and Miskitu. The level of education in the country is variable but generally low relative to Western nations: for instance in our own previous research in the same region of Nicaragua, mean years of education was 6.9 (Boothroyd et al., 2019). Nearly 30% of people live below the poverty line (World Factbook, 2019). The GINI index for Nicaragua is 47.1 and gross national income per capita is around United States $5,900 (World Factbook, 2019). In our rural Nicaraguan field site, media access varied considerably depending on local electricity supply. While internet access was a theoretical possibility in villages with a phone signal, most people were not able to afford smart phones or computers, so television was the main source of visual media for this population at the time of data collection (see Boothroyd et al., 2016).

Our study aimed to address the following research questions:

#### Do Men in These Three Countries Have Similar Body Ideals?

Very little research on men’s body ideals has been carried out in African populations, but there does appear to be a preference for larger bodies compared with those in Britain (e.g., Furnham and Baguma, 1994; Tovée et al., 2006). Nigerian and Ugandan men also desired a body similar to, or slightly larger than their own perceived body size (Maruf et al., 2012; Okoro et al., 2014). Preferences for muscularity are more ambiguous. Ariaal herdsmen in Kenya desired a more muscular body (Campbell et al., 2005), and among Hadza hunter gatherers a more developed upper body was a predictor of positive reputation (Apicella, 2014). Conversely, Ghanaian men showed the least desire for muscularity in comparison to Ukrainian and American men (Frederick et al., 2007). No studies on male bodies have been reported in Nicaragua but a study carried out in the nearby Caribbean island of St Kitts found no differences in men’s ideal fat and muscularity levels relative to men in the United States (Gray and Frederick, 2012). The present study will determine whether the men in our three populations show differences in their ideal male body.

#### Are There Country Differences in Men’s General Body Appreciation?

Previous studies have suggested there may be differences in body appreciation among men across cultural and ethnic groups (Ricciardelli et al., 2007b). For example, Black and Hispanic men in the United States displayed less body image concerns than their White compatriots (Fiske et al., 2014). We postulated that men’s body appreciation would be higher among Ugandan and Nicaraguan men compared with men in the United Kingdom.

#### Does Men’s Media Use and Internalisation Differ Between Countries?

We expected to find significant differences in TV consumption across our three populations due to differences in general media access. We further postulated that media internalisation would differ, with men in the United Kingdom showing higher internalisation than men from Uganda and Nicaragua.

#### What Sociocultural Factors, Including Media and Ethnicity, Predict a Drive for Muscularity, and in Turn, Does the Drive for Muscularity Predict Men’s Body Goals?

There is evidence that aside from media influence, peers and family may also play a role in drive for muscularity (McCabe et al., 2015). We used a Tripartite model to examine whether these sociocultural influences predicted attitudes toward and behaviors around muscularity among men in our three countries. Furthermore, based on previous research that suggests ethnic differences in men’s desire for muscularity, we also included participant ethnicity into the model. While we expected that media and peer influences would predict drive for muscularity among White men in the United Kingdom, we did not make any *a priori* prediction regarding Nicaraguan or Ugandan men.

Employing machine learning algorithms, we also explored if, in turn, muscularity-oriented attitudes and behaviors predicted the likelihood of men from different ethnic groups engaging in body change behaviors.

## Materials and Methods

### Participants

#### Nicaragua

The lead author (TT) had been working in this region for several years on an associated project, so was very familiar with the people and the local culture. Participants were recruited to this study via word of mouth. In the two smaller communities, communal leaders were initially approached who then notified villagers of the researcher’s presence and invited them to participate. In the third larger community, participants were recruited and tested by a local male research assistant trained by TT. A total of 95 men, aged from 15 to 49 (*M* = 24.1 years, *SD* = 7.86) were recruited from three communities around the Pearl Lagoon Basin. In this region of Nicaragua, a man is considered to be a man when he is able to work and provide for his family. Many men are fathers and breadwinners well before the age of 18. Therefore, as with our previous Nicaraguan studies (see Boothroyd et al., 2016; Jucker et al., 2017; Thornborrow et al., 2018; Boothroyd et al., 2019), self-identified men under the age of 18 were not prevented from taking part (*n* = 10). Participants self -identified as belonging to Miskitu (*N* = 68), Mestizo (*N* = 21), and Creole / Garifuna (*N* = 6) ethnic groups. Mean number of years in full time education was 8.9, ranging from 0 to 16 years (*SD* = 3.38). All methods and procedures used in Nicaragua were approved by Durham University Department of Psychology Ethics Committee, reference: 13/17.

#### Uganda

Co-author SM is Ugandan and collected the data in Kampala. Participants were recruited using opportuning sampling and via written invitations sent to all employees (including cleaners, security staff, factory floor workers and management) of a large pharmaceutical manufacturing company. The Ugandan sample consisted of 31 men, aged from 22 to 45 (*M* = 32.1 years, *SD* = 6.31) who belonged to 16 different ethnic groups. However, as these ethnic groups share key characteristics (i.e., Black African background and culture) and all participants were residing in the same urban environment, for the purposes of our analyses we refer to this sample as Black African. Participants had completed an average of 15.1 years of education, ranging from 10 to 22 years (*SD* = 2.76).

#### United Kingdom

Participants were recruited from staff and students using opportunity sampling at the University of Lincoln and from the general community in Lincoln and Leicester. A total of 69 male participants, aged 18 to 45 (*M* = 27.5 years, *SD* = 7.92) were recruited. Nearly two thirds of the sample (*N* = 45) self-identified as of a White ethnic group, and just over a third (*N* = 24) as of a Black ethnic group. The subset of Black males had been living in the United Kingdom or a similar WEIRD country for an average of 10.8 years, ranging from 2 to 30 years (*SD* = 5.45). The number of years in education varied from 6 to 28 years across the sample (*M* = 16.8 years, *SD* = 3.34).

### Measures

#### Demographic Information, Media Exposure and Anthropometrics

Participants self-reported their age, nationality, ethnicity, years of completed full-time education, whether they watched television and, if so, the number of hours they spend watching television in an average week. In the United Kingdom, participants reported all visual media viewed including television, online, Netflix, etc., Participants’ height (cm), weight (kg), chest circumference (cm), and waist circumference (cm) were measured to calculate body mass index (BMI; weight divided by height squared) as an index of body size, and waist-to-chest ratio (WCR; waist circumference in cm divided by chest circumference in cm) as a measure of upper body shape.

#### Men’s Perceived and Ideal Body

**FIGURE 1:**
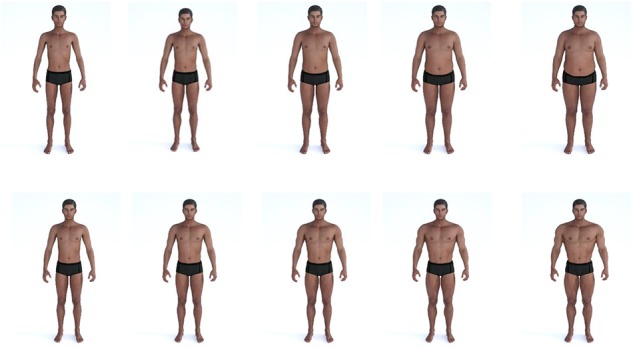
Male Adiposity and Muscularity Scale (MAMS) The images on the top panel depict increase in adiposity The lower panel images depict a comparable increase in Muscularity.

The Male Adiposity and Muscularity Scale (MAMS) was created and utilized to specifically determine participant’s perceived level of muscularity of their current body and their ideal body (see Figure 1). The scale consists of ten computer-generated but realistic images of an ethnically non-White male that vary only by their degree of adiposity or muscularity with all other features remaining identical. The bodies were created in Daz Studio 3D figure modeling software^1^ using the Genesis 2 male model and the Genesis 2 body morphs. In five images the bodies increase in adiposity and represent a range of WCR ratios between 0.85 and 1.01, and five images increase in muscularity and represent WCR ratios between 0.65 and 0.80. Lower WCR values represent a more “v-shaped” torso. Although the scale was primarily designed to measure body perceptions relating to body shape and muscularity, it can also be utilized to assess perceptions of general body size.

#### General Body Appreciation

Men’s general satisfaction with their bodies was measured using the Body Appreciation Scale-2 (BAS-2; Tylka and Wood-Barcalow, 2015). This 10-item scale is rated on a 5-point Likert scale (1 = never, 5 = always), with higher overall or average scores signifying a more positive general body appreciation. It has been used with women and men in both Western and non-Western populations (Tiggemann, 2015; Góngora et al., 2020). A Latin Spanish translation of the scale was constructed for the Nicaraguan part of the study. This version has since been validated (see Góngora et al., 2020). The present sample demonstrated excellent internal reliability (Cronbach’s α = 0.93).

#### Media Belief

Belief in media’s appearance ideals (internalisation) was measured using the Sociocultural Attitudes Toward Appearance Questionnaire-3 (SATAQ-3; Thompson et al., 2004). The scale consists of a total of 30 items rated on a 5-point Likert scale (1 = disagree strongly, 5 = agree strongly). SATAQ-3 has been used in Western (Karazsia and Crowther, 2009; Sánchez-Carracedo et al., 2012) and in non-Western populations (Becker et al., 2011; Chang et al., 2013). In Nicaragua, we also employed the validated Spanish translation of the questionnaire (Sánchez-Carracedo et al., 2012). As there were no magazines at the time of data collection, and the negatively worded statements caused confusion for our rural Nicaraguan participants, we used a modified version of the scale that did not include those items. Participants in the United Kingdom and Uganda responded to all 30 items, but we only used the same 18 items as in the modified version for the analyses. The Cronbach’s alpha for the 18 items in the current sample was excellent (α = 0.90).

#### Drive for Muscularity

We employed McCreary and Sasse’s (2000) Drive for Muscularity Scale (DMS) to measure drive for muscularity. Participants rated 15 items on a 6-point Likert scale, where 1 = never and 6 = always. There are two subscales which assess an attitudinal desire for increased muscle mass (referred to herewith as DMSAtt), and engagement in muscularity-enhancing behaviors (here referred to as DMSBeh). Men tend to score more highly on the scale than women, confirming general gender differences in the salience of the muscular body type (Swami, 2016). In Nicaragua, we also utilized the validated Spanish translation of the questionnaire (Sepulveda et al., 2015). The present sample yielded good reliability (α = 0.86).

#### Body Goals

Men were asked if they were currently trying to lose weight, not trying to change their body weight, trying to gain weight in general, or trying to increase muscle. Participants were allowed to choose more than one option. One Nicaraguan participant said he wanted to lose weight but gain muscle. As this implies desire for lean muscularity, he was coded as “trying to increase muscle.”

#### Family and Peer Influence

Two items from the Perceived Sociocultural Pressures Scale (PSPS; Rohde et al., 2017) were used to assess perceived pressures from friends (“I’ve noticed a strong message from my friends to have a muscular body”) and family (“I’ve noticed a strong message from my family to have a muscular body”). Participants responded using a 5-point Likert rating scale where 1 = never and 5 = always. These two items were translated into Spanish by the lead author (TT). Back translations were made by a bi-lingual Colombian graduate student and then checked by a Nicaraguan school teacher to ensure suitability for a rural Nicaraguan population.

### Procedure

A similar protocol was used for data collection in all three countries. Participants took part individually in a quiet room with a desk. Nicaraguan participants were offered the choice of participating in English or Spanish. In this sample, 53.6% (*n* = 51) participants took part in Spanish. Nicaraguan participants who were under 16 years were tested with a guardian present.

In Nicaragua and Uganda, all demographic and media exposure information was provided verbally by participants, whereas in the United Kingdom sample, this information was provided through written response. Firstly, anthropometric measurements were taken, asking participants to remove footwear and any bulky clothing. Individuals were given the opportunity to take their own body measurements with guidance if they preferred. Next, the MAMS images were presented on a table in a random order, in two rows of five. Participants were asked to select the image that most closely resembled their current body, followed by their desired body. Participants then responded to the following questionnaires: SATAQ-3, PSPS, BAS-2 and the DMS. All questionnaires were provided in the English language in the United Kingdom and in Uganda. In Nicaragua participants were given the option of English or Spanish versions. The entire data collection process took approximately 30 to 45 min per participant. Nicaraguan participants received a payment of £2.50 in local currency for their time. Participants in the Ugandan and United Kingdom samples did not receive any compensation for their time.

## Data Handling and Analyses

As we were interested to ascertain men’s perceptions relating to muscularity rather than simply body size, scoring for participants’ ideal and perceived muscular body selected from the MAMS images was as follows. If a participant picked a body from any of the adipose images, they were given a score of 0 (not a muscular body type). If the least muscular body from the muscularity images was selected, this was coded 1, and so on, with the most muscular body coded as 5. To measure participants’ perceived body size, the same perceived body selected was then coded according to its position on the scale (i.e., 1–5) regardless of adiposity or muscularity.

Item scores for each questionnaire were averaged to obtain a mean score for each participant for body appreciation (BAS-2), media belief (SATAQ), and drive for muscularity (DMS). Country comparisons of means were carried out using one-way between-subject ANOVAs. A criterion of *p* < 0.05 was used for all analyses, and where appropriate, Tukey corrected *post hoc* tests were used for *post hoc* comparisons whilst adjusting for family-wise errors.

Spearman rho correlations were performed to examine the relationships between all main variables. Two separate multiple linear regressions were conducted to ascertain whether sociocultural factors including media and ethnicity predicted men’s muscularity-oriented attitudes (DMSAtt) and behaviors (DMSBeh).

To examine whether a drive for muscularity, sociocultural influences (family, friends, and media) and ethnicity can predict body change behaviors in men, we performed a multinomial logistic regression using body goals as the criterion variable. We used a cross validation approach (James et al., 2013) to split the dataset repeatedly into a training set (80%) used to train (i.e., build) the model and a test set (20%), to validate the prediction of the model. To examine whether the type of prediction analysis affected classification, we used different machine learning classifiers: K-nearest neighbour-(KNN), Random Forests (Breiman, 2001; Rokach and Maimon, 2015), and decision tree to find out which method has the best predictive accuracy based on the yet unseen data set. Although these methods assume the data to be independent, violations of this requirement were not grave in the present application, since our main aim was to assess how well a given model based on measures of sociocultural influences and ethnicity could predict weight status in a set of unseen data (Bali et al., 2016). For this, we applied a 20-fold cross validation to evaluate and supervise the training of the models. All analyses were conducted in R studio computing environment using relevant packages (R Studio Team, 2016).

## Results

### Country Comparisons of Main Variables

Means and standard deviations for the main variables for the whole sample are presented in Table 1.

**TABLE 1:** Means, standard deviations, of main variables and their Spearman’s rank correlations with confidence intervals.

**Variable**	***M***	***SD***	**1**	**2**	**3**	**4**	**5**	**6**	**7**	**8**	**9**
1. TV Hours	13.69	11.08									
2. MAMS-IM	2.35	1.58	0.21**								
			[0.07, 0.34]								
3. BAS	41.76	8.15	−0.24**	−0.23**							
			[−0.37, −0.10]	[−0.36, −0.09]							
4. SATAQ	55.71	21.43	0.16*	0.30**	−0.32**						
			[0.02, 0.30]	[0.16, 0.42]	[−0.44, −0.19]						
5. DMSAtt	22.32	9.96	0.17*	0.23**	−0.04	0.39**					
			[0.03, 0.30]	[0.09, 0.36]	[−0.18, 0.10]	[0.26, 0.50]					
6. DMSBeh	15.71	9.45	0.33**	0.35**	−0.43**	0.50**	0.35**				
			[0.20, 0.45]	[0.22, 0.47]	[−0.54, −0.31]	[0.38, 0.60]	[0.22, 0.47]				
7. BMI	23.90	3.91	0.02	0.10	−0.12	0.04	−0.05	0.17*			
			[−0.12, 0.16]	[−0.05, 0.23]	[−0.25, 0.03]	[−0.10, 0.18]	[−0.19, 0.09]	[0.03, 0.30]			
8. WCR	0.90	0.09	−0.02	−0.17*	0.16*	−0.26**	−0.18*	−0.17*	0.24**		
			[−0.16, 0.12]	[−0.30, −0.03]	[0.02, 0.30]	[−0.39, −0.12]	[−0.31, −0.04]	[−0.30, −0.03]	[0.11, 0.37]		
9. MAMS-PS	2.07	1.10	0.05	0.18*	−0.12	0.07	−0.02	0.25**	0.64**	0.12	
			[−0.09, 0.19]	[0.04, 0.31]	[−0.25, 0.02]	[−0.07, 0.21]	[−0.16, 0.12]	[0.11, 0.38]	[0.55, 0.72]	[−0.02, 0.26]	
10. MAMS-PM	0.95	1.18	−0.00	0.02	0.07	0.03	0.16*	0.17*	−0.09	−0.22**	0.25**
			[−0.14, 0.14]	[−0.12, 0.16]	[−0.07, 0.21]	[−0.11, 0.17]	[0.02, 0.30]	[0.03, 0.30]	[−0.23, 0.05]	[−0.35, −0.08]	[0.11, 0.38]

*M and SD* are used to represent mean and standard deviation, respectively. Values in square brackets indicate the 95% confidence interval for each correlation. *p < 0.05; **p < 0.01. TV Hours, Media use; MAMS-IM, Ideal muscular body; BAS, Body appreciation scale; SATAQ, media belief; DMSAtt, Drive for muscularity – attitude subscale; DMSBeh, Drive for muscularity- behavior subscale; BMI, Body mass index; WCR, waist-to-chest ratio; MAMS-PS, Perceived body size; MAMS-PM, Perceived muscularity.

#### Men’s Actual Body Size and Shape

**FIGURE 2:**
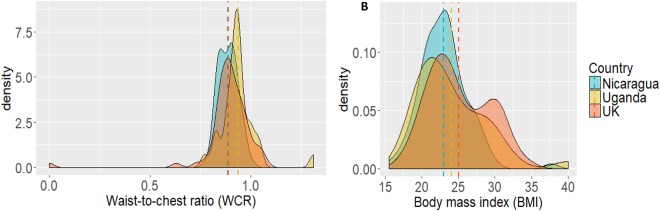
Density of WCR **(A)** and BMI **(B)** by country. The dashed line represents the mean for each country.

The density distributions and means of men’s waist-to-chest ratio (WCR) and body mass index (BMI) by country are presented in Figures 2A,B. Men from the three countries differed on WCR, *F*(2,192) = 3.72, *p* = 0.026, η^2^ = 0.037, with Ugandan men having a less “v” shaped body than men in the United Kingdom and Nicaragua, as indicated by a higher WCR (all *ps* ≤ 0.049). There was no statistically significant difference between United Kingdom and Nicaraguan men (*p* = 0.974). There was also a significant difference in participant BMI across the three countries, *F*(2,192) = 5.64, *p* = 0.004, η^2^ = 0.055, with men in the United Kingdom having a higher BMI than men from Nicaragua (*p* = 0.003). There were no other significant differences (all *p*s > 0.3).

#### Men’s Body Appreciation (BAS)

**FIGURE 3:**
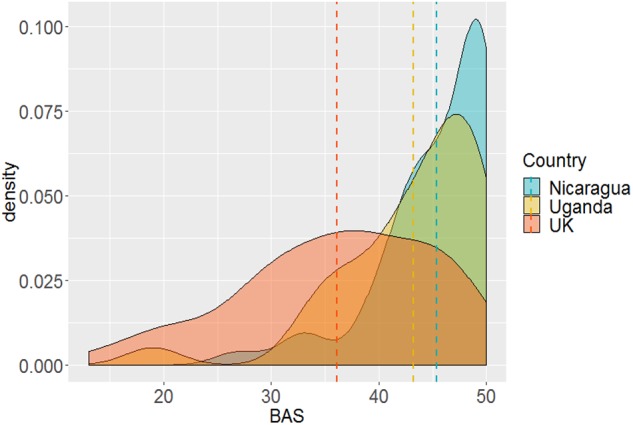
Density of general body appreciation (BAS) by country. Dashed lines represents country means.

The density distributions and means of general body appreciation (BAS scores) by country are shown in Figure 3. Mean BAS score was highest in the Nicaraguan sample (45.4 ± 0.37, Mean ± SEM), lowest for the United Kingdom sample (36.1 ± 0.64), with Ugandan sample mean being intermediate (43.2 ± 0.48). There was a significant main effect of country, *F*(2,192) = 36.23, *p* < 0.001, η^2^ = 0.273. Tukey *post hoc* tests showed that BAS scores were significantly lower in the United Kingdom than both Nicaragua (*p* < 0.001) and Uganda (*p* < 0.001), who did not differ from each other (*p* = 0.280).

#### Men’s Media Use and Media Belief

**FIGURE 4:**
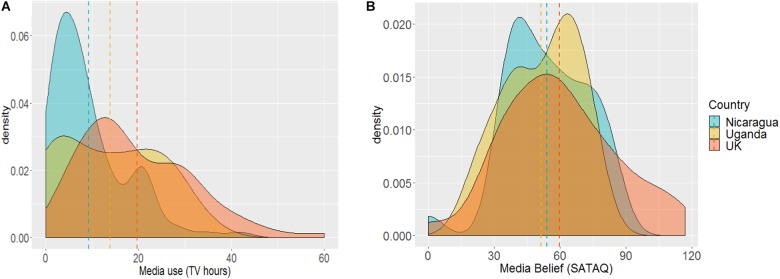
Density of Media Use TV (Hours) **(A)** and Media Belief (SATAQ scores) **(B**) by country. Dashed lines represents country means.

Figures 4A,B plot density distribution and means for media use (TV Hours) and media belief (SATAQ scores) as a function of country. Mean media use, measured as hours per week TV, was higher in the United Kingdom (21 ± 1.13) than Uganda (13.8 ± 0.77) and Nicaragua (9.28 ± 0.58). As expected, there was a significant main effect of country, *F*(2,192) = 19.8, *p* < 0.001, η^2^ = 0.171, with men in the United Kingdom watching more TV than those in Uganda and Nicaragua (all *ps* ≤ 0.014), who did not differ from each other (*p* = 0.150, Figure 4A).

Similarly, there was a significant main effect of country for mean SATAQ scores, *F*(2,187) = 3.26, *p* = 0.041, η^2^ = 0.034, with men in the United Kingdom having greater belief in media than those from Uganda (*p* = 0.048). Nicaraguan men were intermediate but not significantly different from either (all *p*s > 0.1, Figure 4B).

#### Men’s Desired and Perceived Muscularity

**FIGURE 5:**
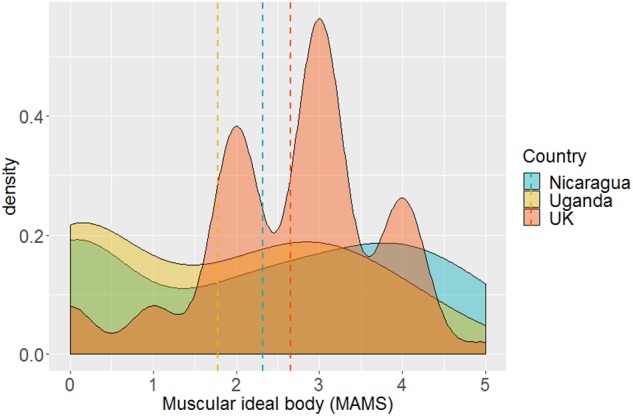
Density distribution of muscular ideal body (MAMS) by country Dashed lines represent country means.

Figure 5 shows density distribution and mean muscular ideal body (MAMS-IM) of all three countries. Country means were: United Kingdom, 2.65 ± 0.08; Uganda, 1.77 ± 0.12; and Nicaragua, 2.32 ± 0.13. Again, there was significant main effect of country, *F*(2,192) = 3.41, *p* = 0.035, η^2^=0.034, with men in the United Kingdom desiring a more muscular body than Ugandan men (*p* = 0.027). Nicaraguan men were intermediate but not significantly different from either (all *p*s > 0.2).

**FIGURE 6:**
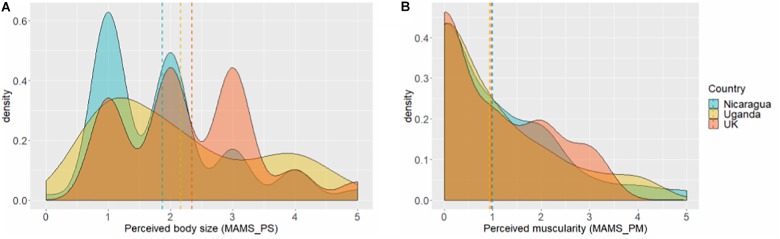
Density of perceived body size (MAMS-PS) **(A)** and perceived Muscularity (MAMS-PM) **(B)** by country. Dashed lines represent country means.

For perceived body size (MAMS-PS, Figure 6A), men in the United Kingdom perceived themselves as larger than Nicaraguan men, *F*(2,192) = 4.73, *p* = 0.009, η^2^ = 0.047, *post hoc p* = 0.008. Ugandan men’s perceived body size was intermediate but not significantly different from either group (*p*s ≥ 0.303). however, there were no significant differences between the three samples for perceived muscularity (MAMS-PM, Figure 6B): United Kingdom, 0.97 ± 0.08; Uganda, 0.94 ± 0.09; and Nicaragua, 0.94 ± 0.09; *F*(2,192) = 0.02, *p* = 0.981, η^2^ = 0.001.

**FIGURE 7:**
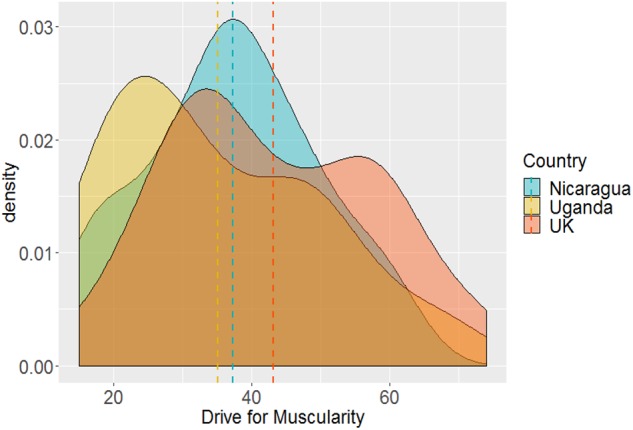
Density distribution of Drive fro Muscularity (DMS) by Country. Dashed lines represents country means.

Figure 7 plots density distribution and mean drive for muscularity (DMS) by country. Mean DMS scores for the three countries were: United Kingdom, 43.1 ± 1.07; Uganda, 35.1 ± 1.09; and Nicaragua, 37.3 ± 0.91. Men in the United Kingdom had greater drive for muscularity than men from Uganda and Nicaragua, *F*(2,185) = 4.78, *p* = 0.009, η^2^ = 0.049 (all *ps* ≤ 0.027) who did not differ from each other (*p* = 0.735).

### Associations Between Men’s Body Perceptions, Drive for Muscularity and Sociocultural Factors

Table 1 shows the means, standard deviations of the main variables and their correlations with confidence intervals. MAMS-IM was significantly and positively correlated with TV hours, SATAQ, DMSAtt, DMSBeh, and MAMS_PS. Perceived body size (MAMS-PS) and men’s actual measured bodies (BMI) were also positively correlated (see Table 1), confirming the MAMS as a reliable measure of body size as well as muscularity.

### Sociocultural Predictors of the Drive for Muscularity

To ascertain what factors predicted men’s attitudes and behaviors around a drive for muscularity, we ran two separate multiple linear regression using the Attitude (DMSAtt) and Behavior (DMSBeh) subscales of the drive for muscularity scale (DMS) as the criterion variables. Following a Tripartite model, we entered 3 measures of sociocultural influences – family, friends (PSPS), and media belief (SATAQ) as predictor variables. As we further postulated that there may be higher drive for muscularity in some ethnic groups (i.e., White British) and we did not know if there would be differences between the other ethnic groups, we also entered participant ethnicity into the model. Table 2 shows the regression coefficients and model summary statistics.

**TABLE 2:** Regression coefficients and model summary.

	*Dependent variable*			
	**Drive for muscularity (Attitude)**		**Drive for muscularity (Behavior)**	
	**(1)**		**(2)**	
	**Estimate**	***SE***	**Estimate**	***SE***
Family	0.519	(0.589)	0.294	(0.455)
Friends	2.102***	(0.524)	0.771^∗^	(0.408)
Media belief	0.074**	(0.035)	0.128***	(0.028)
**Ethnicity (ref: Black African)**				
White British	4.537**	(2.064)	9.743***	(1.597)
Black British	2.740	(2.295)	2.294	(1.838)
Miskitu	4.740**	(1.852)	−4.209***	(1.430)
Mestizo	0.808	(2.422)	–1.770	(1.871)
Black Creole/Garifuna	4.770	(3.748)	2.853	(2.895)
Constant	9.153***	(2.301)	5.488***	(1.789)
Observations	191		188	
*R*^2^	0.255		0.516	
Adjusted *R*^2^	0.222		0.494	
Residual Std. Error	8.389 (df = 182)		6.478 (df = 179)	
*F* Statistic	7.794*** (df = 8; 182)		23.866*** (df = 8; 179)	

*p < 0.1; **p < 0.05; ***p < 0.01.

For the DMSAtt, the regression model was significant [*F*(8,182) = 7.79, *p* < 0.001], with an *R*^2^ of 0.26. Friends (β = 2.11, *p* < 0.001) and media belief (β = 0.07, *p* = 0.036) contributed significantly to the model. Also, belonging to White British (β = 4.54, *p* = 0.029) and Miskitu (β = 4.74, *p* = 0.011) ethnic groups significantly predicted scores on the attitudinal component of the drive for muscularity.

For DMSBeh, the regression model was also significant [*F*(8,179) = 23.89, *p* < 0.001], with an *R*^2^ of 0.52. Of the sociocultural influences, only media belief (β = 0.13, *p* < 0.001) contributed significantly to the model. Again, belonging to White British (β = 9.74, *p* < 0.001) and Miskitu (β = −4.21, *p* = 0.004) ethnic groups significantly predicted scores on the behavioral component of the drive for muscularity.

### Can an Individual’s Body Goal Be Predicted by the Drive for Muscularity and Sociocultural Factors, Including Media and Ethnicity?

To find out what factors predicted men’s body goal, we conducted a multinomial logistic regression using drive for muscularity subscales attitudes (DMSAtt) and behaviors (DMSBeh), ethnicity, measures of sociocultural influences – family, friends, and media belief, as predictor variables. Table 3 shows the model coefficients and associated *p*-values. “Not trying to increase” was used as the reference category. This category combined participant responses of “trying to lose weight” and “not trying to change weight”. Thus, the coefficients compared “Not trying to increase” with “Trying to increase weight,” and with “Trying to increase muscles.”

**TABLE 3:** Regression coefficients and standard error on the outcome variable (body goal).

	***Dependent variable***			
	**Trying to increase weight**		**Trying to increase muscles**	
	**(1)**		**(2)**	
	**Estimate**	***SE***	**Estimate**	***SE***
Friends	0.024	(0.217)	0.265	(0.212)
Family	0.099	(0.247)	–0.130	(0.219)
SATAQ	−0.048**	(0.019)	–0.013	(0.017)
**Ethnicity (ref: Black African)**				
Black British	–0.157	(0.991)	–0.223	(1.573)
Black Creole/Garifuna	−14.062***	(0.00000)	3.062**	(1.445)
Mestizo	–0.243	(1.150)	2.584**	(1.279)
Miskitu	0.482	(0.798)	1.809	(1.155)
White British	–1.742	(1.303)	–0.276	(1.326)
DMSAtt	0.141***	(0.035)	0.087**	(0.034)
DMSBeh	–0.012	(0.046)	0.078^∗^	(0.042)
Constant	−2.386**	(1.076)	−5.911***	(1.537)
Akaike Inf. Crit.	237.829		237.829	

*p < 0.1; **p < 0.05; ***p < 0.01.

For the predictor variable drive for muscularity attitudes subscale (DMSAtt), the log odds of body goal with “Trying to increase weight” versus “Not trying to increase” will increase by 0.141 (*p* < 0.001) whilst a one unit increase in DMSAtt will increase the log odds of “Trying to increase muscles” versus “Not trying to increase” will increase by 0.087 (*p* = 0.035). A one-unit change in media belief decreased the log odds of body goal with “Trying to increase weight” versus “Not trying to increase” by −0.048 (*p* = 0.011). Compared with the Black African ethnic group (the reference category), belonging to the Black Creole/Garifuna ethnic group decreased the log odds of body goal with “Trying to increase weight” versus “Not trying to increase” by −14.062 (*p* < 0.001) whilst the log odds of body goal with “Trying to increase muscles” versus “Not trying to increase” is increased by 3.062 (*p* = 0.034). The log odds of body goal with “Trying to increase muscles” versus “Not trying to increase” will increase if belonging to the Mestizo ethnic group by 2.584 (*p* = 0.043).

Overall, the multinomial regression analysis suggests that body goals can be predicted by belief in media, drive for muscularity (attitudinal dimension) and belonging to certain ethnic groups. Our analysis showed that Black Creole/Garifuna and Mestizo men are more likely to want to actively increase their body’s muscularity. Furthermore, Black Creole/Garifuna men are less likely to want to increase general body weight.

The confusion matrix is presented in Table 4 and illustrates how accurate the model predicts the correct classes in the test data. The overall model accuracy for the training dataset is 71%. When the model was applied to yet unseen data, the accuracy improved to 72%, indicating no overfitting in the training dataset.

**TABLE 4:** Confusion matrix showing the proportion of correct and incorrect predictions broken down by body goal (Test data).

**Predicted**					
**Actual**		**WO**	**W1**	**W2**	**Total**
	**WO**	29	1	0	30
	**W1**	2	1	1	4
	**W2**	3	0	1	4
	**Total**	34	2	2	**38**

*The diagonal shows the number of observations that have been correctly predicted. **W0** = “Not trying to increase.” **W1** = “Trying to increase weight.” **W2** = “Trying to increase muscles.”*

**FIGURE 8:**
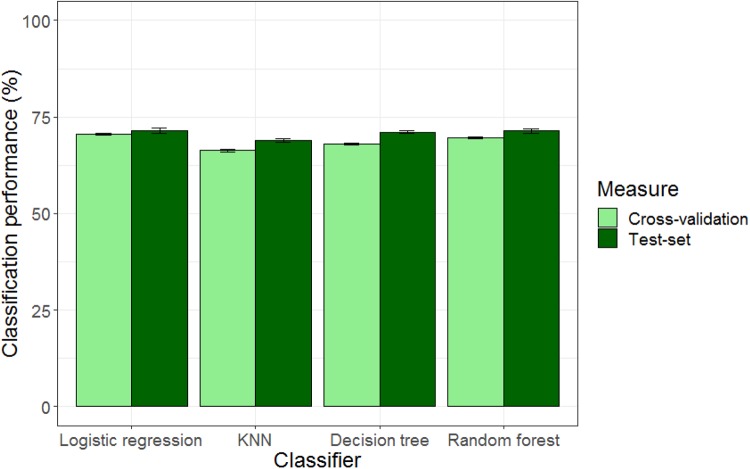
Model predictive accuracy across several classifiers for 20-fold cross-validation (80%) and test set (20%).

Figure 8 shows classification performance for different machine learning classifiers. Logistic regression yielded the best classification accuracy of 71.4%, while Random forests, and Decision Tree, and KNN achieved accuracies of 71.3, 71, and 69% respectively.

## Discussion

The present study investigated media influence on men’s body image in two non-White, non-WEIRD populations in two of the most understudied regions of the world, Africa and Central America. We compared the body ideals and body image of ethnically diverse samples of men in Nicaragua, Uganda, and the United Kingdom. Using a Tripartite framework, we further investigated the role of ethnicity, media and other sociocultural influences in predicting men’s drive for muscularity and in turn, their body change behaviors.

### Country Comparisons of Men’s Body Ideals and Body Image

We found significant country level differences in men’s ideal body. Men in the United Kingdom had a significantly more muscular ideal body than Ugandan men who showed the lowest desire for a muscular body. This is consistent with results from Frederick et al.’s (2007) study which found that Ghanaian men had less desire for a muscular body than men in the United States and Ukraine. The authors suggested that as Ghanaian men already have more muscle they may already be much nearer to their ideal body. The Nicaraguan men’s ideal body was not significantly less muscular than that of the men in the United Kingdom which is consistent with research which found that Black Caribbean men and men in the United States preferred a similarly muscular body (Gray and Frederick, 2012). Country differences in the drive for muscularity followed a similar pattern to differences in ideal muscularity, with Ugandan men having the least drive for muscularity compared with men in the United Kingdom.

There were also some significant country differences in participant body shape and size that partly reflected the differences found in men’s body ideals. The Ugandan participants had a significantly less “v” shaped body than the men in the United Kingdom and Nicaraguan participants, but there was no difference between countries for participant BMI. The differences in participants’ actual body shapes in the three countries may reflect differences in diet and lifestyle (Miller et al., 1990; Nelson and Tucker, 1996; Verheggen et al., 2016). For example, the Nicaraguan men in our rural sample are likely to be more physically active and have a different diet as compared to the men in the United Kingdom (Jucker et al., 2017). Additionally, different ethnic groups have different patterns of fat distribution on their body (Wells et al., 2008, 2012) and as a result, may have different body shapes and/or BMI cut-offs for health (Wildman et al., 2004; Lear et al., 2010). For example, the boundary for the overweight category is 23 and not 25 for people of Chinese or Indian descent (Choo, 2002; Shiwaku et al., 2004; Kesavachandran et al., 2012). This has implications for what is perceived as a healthy or desirable body size and shape. Many studies have argued that ideal body size and shape reflect what is perceived to be the healthiest and fittest size and shape for a particular environment (Thornhill and Grammer, 1999; Tovée et al., 2006). Thus, it is possible that the ideal body size and shape might differ across ethnic groups reflecting general differences in physiology.

Body appreciation was on average significantly higher among Nicaraguan and Ugandan men than in men in the United Kingdom. Previous comparisons of body appreciation within Western contexts have found higher levels of body appreciation and satisfaction among ethnic minority groups which may suggest a degree of cultural “shielding” from mainstream Western ideals (Wildes et al., 2001; Rodgers et al., 2018). Interestingly, the Nicaraguan men’s mean BAS scores were higher than those of Mexican, Colombian and Argentinian adolescents (Góngora et al., 2020). There are factors that differentiate Nicaragua from these countries, and which may explain these differences. Firstly, Mexico and Argentina could be considered closer to WEIRD populations in many respects. Even if not strictly “Western,” they are influenced by White United States and European cultural identities so people may aspire to more Western appearance standards (Meehan and Katzman, 2001). Indeed, research among women from Argentina and Brazil has found that their appearance satisfaction was similarly low to that of United States women (Forbes et al., 2012). Secondly, participants in the aforementioned studies were more likely to be of Spanish European background, while the majority of our Nicaraguan participants were from ethnic groups with Indigenous and Black African backgrounds: Research suggests that men from non-White ethnic groups often have higher body satisfaction than Hispanic or White men (Ricciardelli et al., 2007b; Kelly et al., 2015).

### Sociocultural Predictors of the Drive for Muscularity and Body Change Behaviors

Across the sample, there were significant associations between media measures and men’s body image, such that more TV viewing and greater media internalisation were associated with both a more muscular ideal body and lower general body appreciation, consistent with previous research in Western (Morrison et al., 2003; Cramblitt and Pritchard, 2013; Pritchard and Cramblitt, 2014) and non-Western populations (Chang et al., 2013). This permitted us to use linear regression models based on a Tripartite influence model to examine which sociocultural factors, including media, predicted men’s drive for muscularity. As in previous studies using a Tripartite framework (Girard et al., 2017), we found that sociocultural influences media belief and peers significantly predicted men’s attitudinal drive for muscularity. Media belief alone predicted scores on the behavioral dimension of the scale. There were also differences across ethnic groups. Belonging to a Black African or Black Creole/Garifuna ethnic group was not associated with a higher drive for muscularity. This is consistent with a study which suggested that Black Caribbean men did not have a higher desire for muscularity than an ethnically mixed United States sample (Gray and Frederick, 2012). By contrast, another study found that in the United Kingdom, Black men had higher drive for muscularity than White men (Swami, 2016). The author suggested that this may because Black British men appropriate their muscularity as a way of negotiating power inequalities, or because they have internalized Western stereotyped tropes of Blackness that emphasise the physicality or “athleticism” of the black male body (Swami, 2016). However, in the same study, stronger identification with traditional cultural values among all participants was associated with lower drive for muscularity, suggesting that men who maintain their own cultural or ethnic group identity may feel less inclined to conform to mainstream (i.e., White) appearance standards (Swami, 2016). Our results would seem to support these contrasting findings: ethnically Black men from the non-White, non-WEIRD nations sampled demonstrated less desire to strive for a muscular ideal body type than those who live in a predominantly White, Westernized nation, possibly because they experience less pressure to aspire to both Western body ideals and stereotyped constructions of Black masculinity.

To further investigate whether a drive for muscularity, family, friends and media could predict likelihood of engaging in body change behaviors, we used multinomial logistic regression and applied machine learning classifiers to evaluate how well a given model could predict body goals in a set of yet unseen data. We found that men’s body goals could be predicted by greater belief in media, high drive for muscularity (attitudinal dimension) and belonging to certain ethnic groups. Importantly, we found that Black Creole/Garifuna and Mestizo men were more likely to want to increase muscularity compared to Black African men. Furthermore, Black Creole / Garifuna men were less likely to want to increase general body weight. It is interesting to note that although Black Creole / Garifuna men were more likely to report a body goal of trying to increase their muscle than both Black British and Black Ugandan men, belonging to Black Creole / Garifuna ethnic groups was not associated with a drive for muscularity, i.e., these men did not appear to feel psychological pressure to achieve a muscular body type. Previous studies looking at body size concerns among Black males have also produced mixed findings. In the United States, Black men had either higher body satisfaction than other ethnic groups or were no different from the other groups. However, Black adolescents have high levels of unhealthy weight control behaviors and dieting (e.g., Doris et al., 2015; Kelly et al., 2015; Rodgers et al., 2017).

### Mechanisms Explaining Sociocultural Influences on Men’s Body Image

Differences in body ideals can potentially arise due to a change in the size or shape of the bodies an individual is viewing (i.e., a change in their visual diet). This is because it is hypothesized that we judge bodies with reference to an internal template, which is based on all the bodies we have seen over the course of our lifetime with a bias toward the most recently viewed (Winkler and Rhodes, 2005; Cornelissen et al., 2013). So, for example, viewing large numbers of fat bodies would shift the observer’s ideal toward a higher BMI (Robinson and Kirkham, 2014; Oldham and Robinson, 2016). Thus, the increased proportion of muscular and lean bodies in the media viewed by our participants may shift their ideal to reflect this change in visual diet. We found significant positive associations between television viewing time, media belief and men’s ideal muscularity, suggesting that the media content our participants are watching is to some degree appearance focused: indeed many participants reported watching sports and action movies, genres that would likely contain a high proportion of athletic or muscular male bodies.

An alternative explanation is based not on changes in visual content, but on changes in how different sizes and shapes are valued (visual valency) (Boothroyd et al., 2012). The presentation of muscular, lean bodies as being indicative of high status in the media propagates this as an ideal in Western countries and the spread of Western media across the world disseminates this message into non-WEIRD populations. Furthermore, pre-existing cultural constructions of maleness and masculinity may already incorporate a muscular body type and so men seek out media content that validates and reinforces them. Certainly, participants in this study reported watching mainly action movies, sports content and news programmes - genres that tend to contain a high proportion of stereotypical representations of masculinity, including body types and behaviors.

In addition to media pressure to conform to a lean and muscular ideal, peer and family influences may also shape men’s body ideals and contribute to body image concerns (Rodgers et al., 2015). For example, in the United Kingdom it is suggested that the strong drive for muscularity comes from peer pressure and the desire for social status (Swami, 2016). A similar drive for social dominance, fitness and sporting performance is found in adolescent boys and young men in Tonga and Fiji (Ricciardelli et al., 2007a). This suggests a modulating effect of family and peers on the development of body image and body concerns, and the influence of cultural differences between our three groups of men.

Other mechanisms such as social comparison may also be involved in a drive for muscularity (Smolak and Stein, 2006; Karazsia and Crowther, 2009). Social comparison theory (Festinger, 1954) would suggest that exposure to the image of a body (whether in real life or through the media) that is thinner or more muscular than they believe themselves to be would represent an aspirational stimulus which should be the cue for upward social comparison. The larger the difference between what they believe themselves to be and the size and shape of the aspirational stimulus, the stronger the pressure. The presentation of lean muscular bodies in the media as a high status, aspirational ideal might thus induce a drive for muscularity. There is considerable evidence consistent with such a social comparison explanation for the proposed effect of the media on body perception and the drive for muscularity in men in the United Kingdom and the United States (e.g., Smolak and Stein, 2006; Karazsia and Crowther, 2009; Galioto and Crowther, 2013). However, the results from our study are more equivocal. The Ugandan participants had the lowest actual muscularity, which would predict that the difference between the media ideals and their own bodies would be the largest. They should therefore have the strongest drive for muscularity, but this does not seem to be the case.

### Study Strengths and Limitations, Future Research

Our research has addressed a critical gap in the body image literature by focusing on men’s body image and related body change behaviors in two non-WEIRD populations, Uganda and Nicaragua. The study has several other notable strengths. Firstly, we sampled men of Black African descent in three different settings, sub-Saharan Africa, Central America and the United Kingdom. Secondly, to the best of our knowledge, this is the first study to sample men of Miskitu ethnicity. Finally, we have also taken a more holistic cross-cultural approach, and considered perceptual and attitudinal aspects of men’s body image.

The study also has several key strengths relating to the methodologies used to assess the perceptual and attitudinal components of men’s body image. We employed well-established, validated self-report questionnaires to measure drive for muscularity, body satisfaction, media belief, and the influence of family and peers. Although these psychometric measures were developed and are predominantly utilized in WEIRD populations, good levels of internal consistency across our sample suggest they were also suitable for use among men in our non-WEIRD contexts. Furthermore, we utilized novel visual stimuli, the Male Adiposity and Muscularity Scale (MAMS), to determine men’s perceptions of their current body and their desire for a muscular ideal body. The scale was specifically designed using ethnically non-White male figures to improve ecological validity and enhance participants’ ability to identify with the images and thus produce meaningful data. High correlations between men’s actual and MAMS perceived body size attest to its construct validity.

While this study has considerable strengths, there were some limitations. Sampling in the two non-WEIRD nations differed somewhat, resulting in a more urbanized Ugandan sample relative to the Nicaraguan sample. Furthermore, we did not include a measure of socioeconomic status (SES) in our analyses, even though evidence suggests that this may be a factor contributing to cross-cultural differences in levels of body dissatisfaction (Swami et al., 2010). We did collect information on participants’ income, but these data were rather problematic for several reasons. Firstly, almost 20% of participants were students and reported having very little or no income. Secondly, the incidence of students was not the same across the three country samples. Lastly, we believe that the responses to the income questions may be rather unreliable, at least in the two non-WEIRD samples. It was observed that in these locations some participants were quite reticent about divulging their earnings, while others appeared to exaggerate them in front of the Western researcher. Additionally, it is problematic to simply compare an income total across countries: earning $1,000 in Nicaragua is not the same as earning the equivalent in the United Kingdom: Such a salary would be considered reflective of a high socioeconomic status within Nicaragua, but not in the United Kingdom. We would strongly recommend that future research incorporates a measure that usefully captures socioeconomic status of participants across a variety of cultural contexts. A relatively small sample was recruited in Uganda, compared to the United Kingdom and Nicaraguan groups, resulting in reduced power to detect statistically significant differences between groups. Further, our participants in Uganda all self-identified as Black African (even though tribal affiliation varied considerably), while those in Nicaragua were of several distinct ethnic groups. However, this was anticipated and so ethnicity was included as a variable in our within-participant analyses. Additionally, many of the Black men in our United Kingdom sample reported being born in a range of African countries, and we did not measure acculturation other than the number of years of residence in the United Kingdom. Nevertheless, a majority of this sample had lived in the United Kingdom (or other Western country) for most of their adult lives, and research suggests that living in a Western nation for a number of years is associated with more Western-like body ideals (Tovée et al., 2006). The present study was cross-sectional, meaning we cannot draw causal inferences from our findings. Overall, correlational studies tend to show small effect sizes of media influence on body image, suggesting that media alone may not explain negative body image or related outcomes (Levine and Murnen, 2009). However, experimental evidence shows that exposure to idealized media imagery shifts viewers’ own body ideals in the same direction in both WEIRD (Boothroyd et al., 2012) and non-WEIRD samples (Boothroyd et al., 2019), and perceived media pressure precedes body dissatisfaction and unhealthy behavioral outcomes (Barlett et al., 2008). However, thus far there is scant experimental research of this kind carried out with culturally diverse samples outside of Western contexts (Blond, 2008). Future research therefore should include experimental and longitudinal studies in non-WEIRD populations to assess the influence of media beyond shaping body ideals and investigate its role in negative body image and related behavioral outcomes (Holmqvist and Frisén, 2010).

We also acknowledge that media use is changing, especially in the non-WEIRD countries sampled here. However at the time of data collection, TV viewing was the only source of visual imagery for our Nicaraguan participants – very few had smartphones or regular access to the internet. Future research is needed to measure usage of these forms of media and identify their effect on body image among populations in non-WEIRD contexts with the aim of ameliorating negative effects currently witnessed among young people in Western populations (e.g., Fardouly and Vartanian, 2016).

Finally, the limited number of body sizes and shapes presented in the MAMS may have meant that participants selected an “approximate” representation of their body ideals. Future studies would benefit from using methods that allow participants to create “bespoke” bodies in 3D to obtain more accurate representations of their body perceptions (e.g., Crossley et al., 2012; Thornborrow et al., 2018).

## Conclusion

The present study investigated men’s body ideals and body image in two non-WEIRD, non-White populations, Uganda (Africa) and Nicaragua (Central America), and compared them with those of men in the United Kingdom. Overall, men in our non-WEIRD samples displayed less desire for muscularity and less body image concerns than men in our WEIRD sample. Furthermore we have demonstrated that media and peer pressure are associated with men’s body image and related body change behaviors in diverse non-White ethnic groups.

## Data Availability Statement

The datasets generated for this study are available on request to the corresponding author.

## Ethics Statement

The study was granted ethical approval by the University of Lincoln’s School of Psychology Research Ethics Committee on the 12th December 2017 (PSY1718346).

## Author Contributions

All authors were involved throughout the study, wrote the manuscript, and approved the final manuscript. TT, MT, and LB designed and conceived the study. MT and TT created the MAMS visual stimuli. TT, SM, and TO collected the data. TO and TT performed the statistical analyses.

## Conflict of Interest

The authors declare that the research was conducted in the absence of any commercial or financial relationships that could be construed as a potential conflict of interest.

## Footnotes

1 www.Daz3D.com
